# Tai-Chi-Chuan Exercise Improves Pulmonary Function and Decreases Exhaled Nitric Oxide Level in Both Asthmatic and Nonasthmatic Children and Improves Quality of Life in Children with Asthma

**DOI:** 10.1155/2017/6287642

**Published:** 2017-04-13

**Authors:** Hsin-Chia Lin, Hao-Pai Lin, Hsin-Hui Yu, Li-Chieh Wang, Jyh-Hong Lee, Yu-Tsan Lin, Yao-Hsu Yang, Pei-Yi Li, Wei-Zen Sun, Bor-Luen Chiang

**Affiliations:** ^1^Department of Pediatrics, National Taiwan University Hospital, Taipei, Taiwan; ^2^Taiwan Tai Chi & Wushu Academy, Taipei, Taiwan; ^3^Department of Anesthesiology, National Taiwan University Hospital, Taipei, Taiwan; ^4^Department of Medical Research, National Taiwan University Hospital, Taipei, Taiwan

## Abstract

Tai-Chi-Chuan (TCC) is an exercise of low-to-moderate intensity which is suitable for asthmatic patients. The aim of our study is to investigate improvements of the lung function, airway inflammation, and quality of life of asthmatic children after TCC. Participants included sixty-one elementary school students and they were divided into asthmatic (*n* = 29) and nonasthmatic (*n* = 32) groups by the International Study of Asthma and Allergies in Childhood (ISAAC) questionnaire. Among them, 20 asthmatic and 18 nonasthmatic children volunteered to participate in a 60-minute TCC exercise weekly for 12 weeks. Baseline and postintervention assessments included forced expiratory volume in one second (FEV1), forced vital capacity (FVC), peak expiratory flow rate (PEFR), fractional exhaled nitric oxide (FeNO) level, and Standardised Pediatric Asthma Quality of Life Questionnaire (PAQLQ(S)). After intervention, the level of FeNO decreased significantly; PEFR and the FEV1/FVC also improved significantly in both asthmatic group and nonasthmatic group after TCC. The asthmatic children also had improved quality of life after TCC. The results indicated that TCC could improve the pulmonary function and decrease airway inflammation in both children with mild asthma and those without asthma. It also improves quality of life in mild asthmatic children. Nevertheless, further studies are required to determine the effect of TCC on children with moderate-to-severe asthma.

## 1. Introduction

Asthma is the most common chronic disease among children in the world [1]. It is characterized by chronic inflammation of the airways, airway hyperresponsiveness, and remodeling. Due to recurring cough, wheezing, breathlessness, and chest tightness, asthma impacts the quality of life of individuals greatly, even under current medical treatment and environmental control in adults or in children. The prevalence of asthma among school age children was estimated to be 7.4% in central Taiwan in 2009 [2].

Although exercise is beneficial with regard to asthma control, exercise is a common trigger for the asthma symptom of bronchospasm. For this reason, asthmatic children may avoid vigorous activity due to concerns regarding the worsening of asthma symptoms or deconditioning due to inactivity. This might result in further reduced physical fitness and social well-being [3]. Exercise that has a minimal impact regarding exercise-induced bronchospasm such as swimming is better tolerated among asthmatic children [3, 4]. Approximately 20 to 30 minutes of exercise at 60% to 85% of maximum heart rate four or five times a week is recommended for asthmatic patients [5]. A recent review demonstrated that physical training showed significant improvements in maximum oxygen uptake, though no effects were observed in other measures of pulmonary function [6]. Recent studies have shown that yoga exercise three times a week for 10 weeks could improve quality of life and decrease asthmatic symptoms in women with mild-to-moderately persistent asthma [7].

Tai-Chi-Chuan (TCC) exercise is often referred to as “meditation through movement,” incorporating elements of balance, postural alignment, and concentration [8]. It is an exercise that combines deep diaphragmatic breathing and relaxation with many fundamental postures, which is suitable for asthmatic patients. An increased oxygen uptake up to 50% of the peak oxygen consumption and heart rate within 58% of the heart rate range were noted during the TCC sessions, suggesting that TCC is a moderate-intensity exercise that is aerobic in nature [9, 10]. Compared to other exercises of an equal intensity, TCC was shown to have a significantly lower ventilatory equivalent (V_E_/V_O2 MAX_) [11]. TCC exercise is beneficial to cardiopulmonary function, mental control, flexibility, and balance control [12]. Studies also showed that TCC had an anti-inflammatory effect, including reducing monocytes counts, enhancing CD4+/CD8+ T cells ratio, and increasing regulatory T cells [13]. Enhanced immune function after TCC exercise has also been observed, including increases in the levels of IgG, increasing the numbers and activity of natural killer cells [12]. Our previous small-scale study showed that pulmonary function significantly improved in asthmatic children after 3 months of TCC, but the improvement in symptoms was not significantly different between the TCC group (15 children) and control group (15 children) [14]. The aim of this study is to investigate the differences regarding the lung function, airway inflammation, and quality of life of asthmatic children after TCC exercise.

## 2. Materials and Methods

### 2.1. Study Subjects and TCC Exercise

We recruited 61 students (29 asthmatic children and 32 nonasthmatic children) from an elementary school in Taipei City. Subjects with congenital heart disease, chronic cardiopulmonary disease, rheumatic or autoimmune disease, neuromuscular disease, and other systemic diseases were excluded. Asthma was initially screened through the International Study of Asthma and Allergies in Childhood (ISAAC) written questionnaire translated in Chinese [15]. The diagnosis of asthma was further confirmed by pediatric allergist/immunologists. All the asthmatic participants had mild asthma without taking any controller medication. Informed consent was obtained from each participant. This study has been approved by the Institutional Review Board of National Taiwan University Hospital.

**Figure 1:**
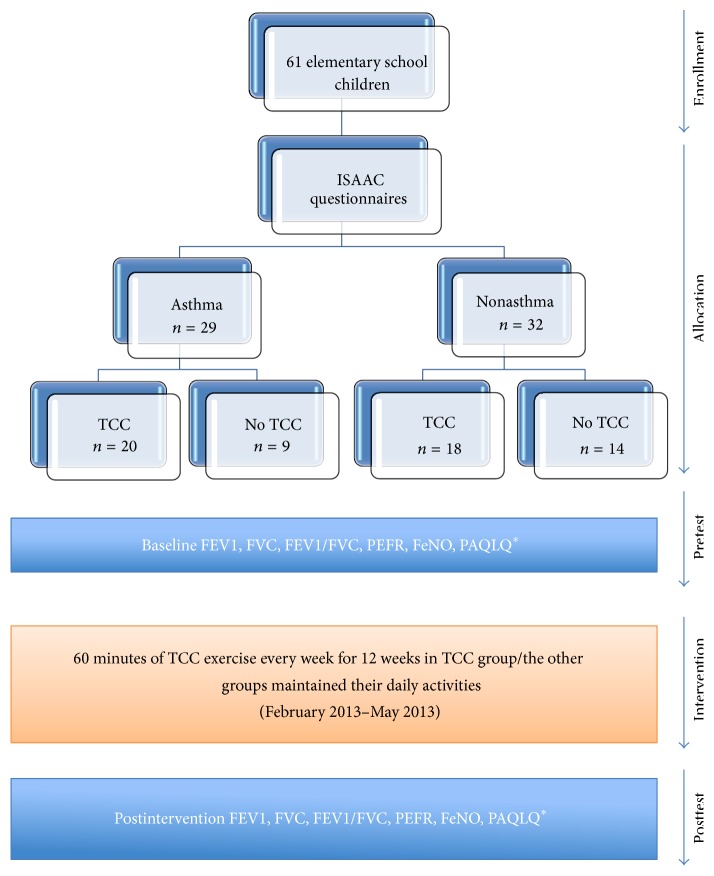
^*∗*^Among asthma group, only 18 in TCC group and 5 in the no TCC group completed the PAQLQ questionnaire.

Among the recruited students, 20 asthmatic and 18 nonasthmatic children volunteered to participate in a 60-minute TCC exercise class once a week for 12 consecutive weeks. Subjects in the TCC training group learned to perform Chen-style TCC standardised movements under the guidance of two TCC teachers (P.-Y. Li and C.-H. Sun) at school. Children were asked to practice TCC exercise assisted by videos daily (Figure 1). Non-TCC training participants were instructed not to begin any new activities but to maintain their daily activities.

Given the limited time frame, the TCC course was specifically designed as a therapy for asthmatic children, under the following points: being able to draw and hold the attention of children; to improve body flexibility and coordination; to increase muscle power (strength and stamina) of the lower extremities; to improve cardiovascular function through mostly moderate-intensity exercise, with about ten minutes of higher-intensity activity; and to develop attentiveness to posture and breath (breathing methods). Each session followed a sequence of warming-up in circular movements, stretching exercises, TCC walking drills, and TCC “opening and closing” movements in stationary positions.

### 2.2. Measurements

All of the study subjects were evaluated at the entry and at the end of the study for lung function, fractional exhaled nitric oxide (FeNO), and quality of life, as assessed by the Pediatric Asthma Quality of Life Questionnaire (PAQLQ). The PAQLQ was completed by 18 asthmatic TCC and 5 asthmatic non-TCC subjects.

#### 2.2.1. Lung Function Test

Forced expiratory volume in one second (FEV1), forced vital capacity (FVC), and FEV1/FVC ratio were measured using Micro Medical Super Spiro spirometer in resting status. Peak expiratory flow rate (PEFR) was assayed using a peak flow meter. Data of FEV1, FVC, and PEFR are expressed as the percentage of the predicted value specific for Taiwanese children. None of the participants had taken any short-acting bronchodilator within 4 hours of undergoing spirometry.

#### 2.2.2. Fractional Exhaled Nitric Oxide (FeNO)

Fractional exhaled nitric oxide (FeNO) concentrations in exhaled breath are a widely used noninvasive marker of airway inflammation in asthma [16]. FeNO was measured using a NIOX MINO machine before and after the 12-week TCC training. It was measured before spirometric maneuvers, at an exhaled rate of 50 ml/second maintained within 10% for more than 6 seconds, and with an oral pressure of 5 to 20 cm H_2_O to ensure velum closure. Results are expressed as the NO concentration in ppb (equivalent to nanoliters/liter) based on the mean of two or three values within 10% [17]. Subjects were informed to have a low nitrogen diet prior to the exam to minimize confounders [18].

#### 2.2.3. Standardised Pediatric Asthma Quality of Life Questionnaire (PAQLQ(S))

We used the traditional Chinese version of PAQLQ(S) developed in Taiwan, which included 23 questions in 3 domains (symptoms, activity limitation, and emotional function) [19]. The activity domain contains 3 “patient-specific” questions. Children were asked to think about how they have been during the previous week and to respond to each of the 23 questions on a 7-point scale (7 = not bothered at all; 1 = extremely bothered). The overall PAQLQ score was the mean of all 23 responses and the individual domain scores were the means of the items in those domains [20]. The measurement sensitivity and validity of the PAQLQ have been validated in several studies for different countries. A change in score greater than 0.5 on the 7-point scale can be considered clinically important [21–24].

### 2.3. Statistical Analysis

Data were compared between groups using the Kruskal-Wallis test or the Mann–Whitney *U* test for continuous variables. The paired *t*-test or Wilcoxon matched-pairs signed-rank test was used to compare the parameters of continuous variables before and after TCC. Stepwise multiple linear regression models were used to identify the effects of the variables of asthma, TCC, gender, age, weight, height, and baseline values, on outcome changes in lung function (FEV1, FVC, FEV1/FVC, or PEF) and airway inflammation (FeNO). All of these analyses were conducted using SPSS statistical software, version 20 (IBM), and GraphPad Prism software, version 6. A significance level of 0.05 was used for statistical comparisons.

## 3. Results

**Figure 2:**
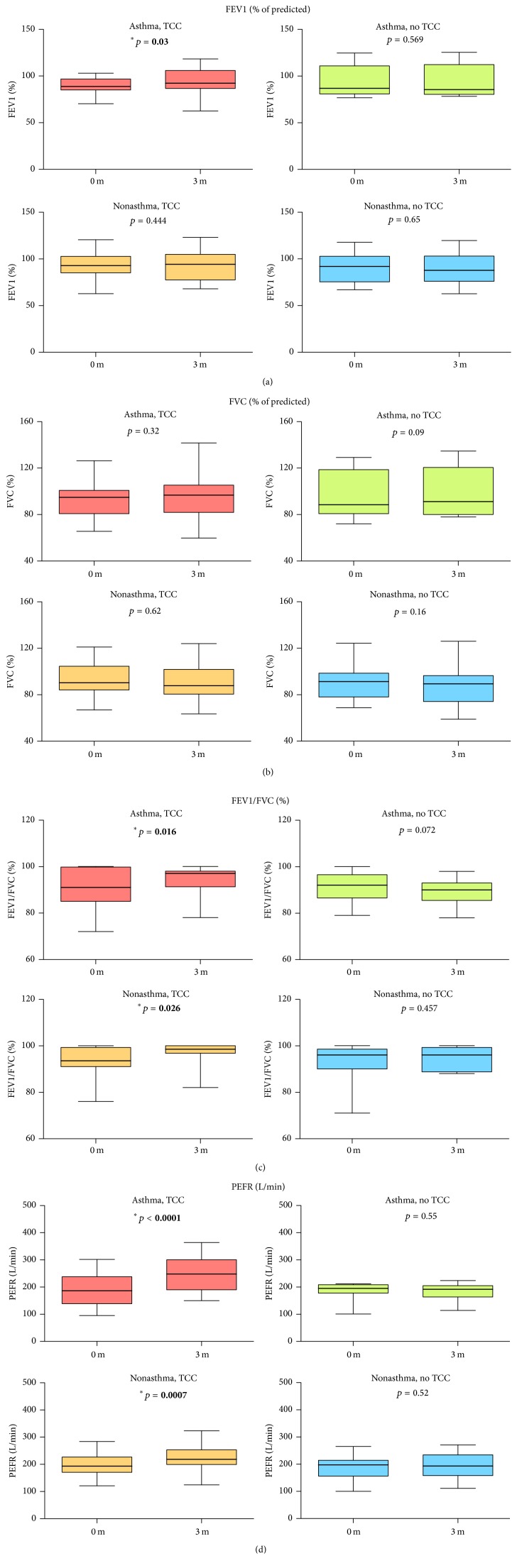
Comparison between pulmonary function at baseline and that after 3 months in each group. Horizontal thick bars indicate median values and box indicates the interquartile range (IQR); upper and lower horizontal bars indicate maximum and minimum values.

**Figure 3:**
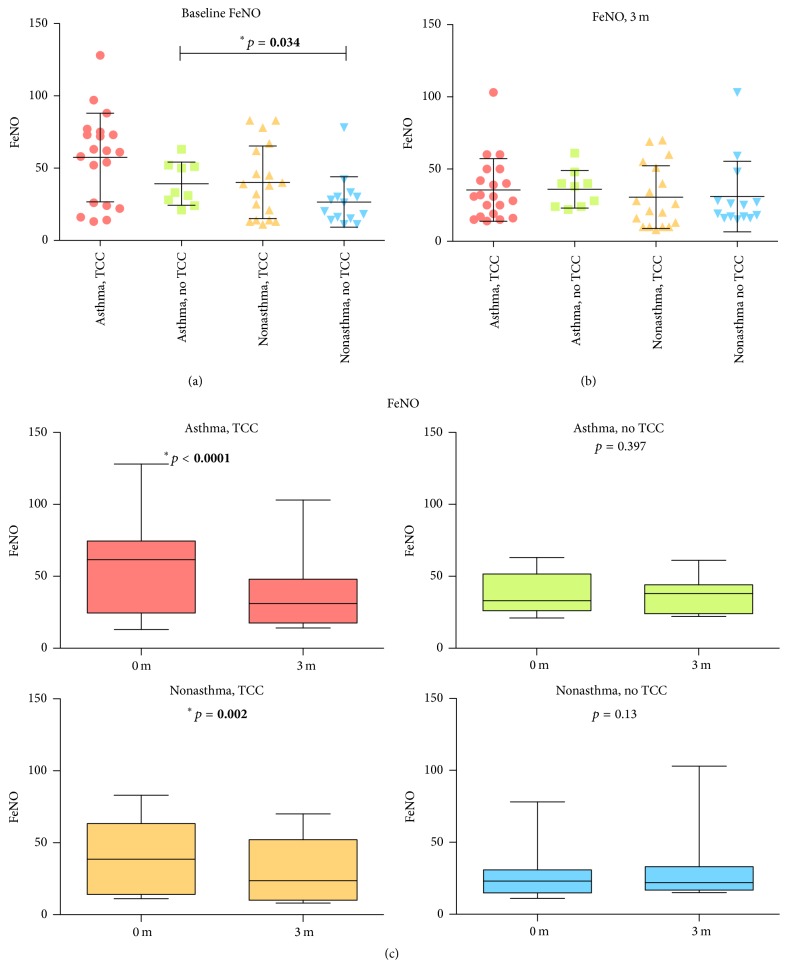
(a) Baseline FeNO level between 4 groups; (b) FeNO level 3 months later between 4 groups; (c) FeNO level before and after 3 months.

**Figure 4:**
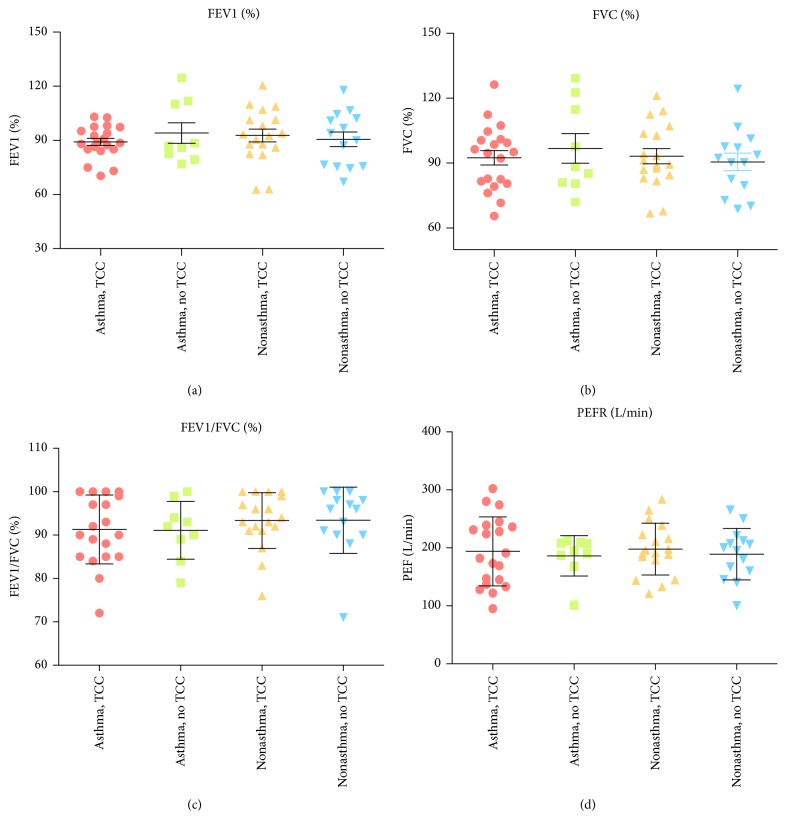
Comparison of baseline pulmonary function between 4 groups.

**Figure 5:**
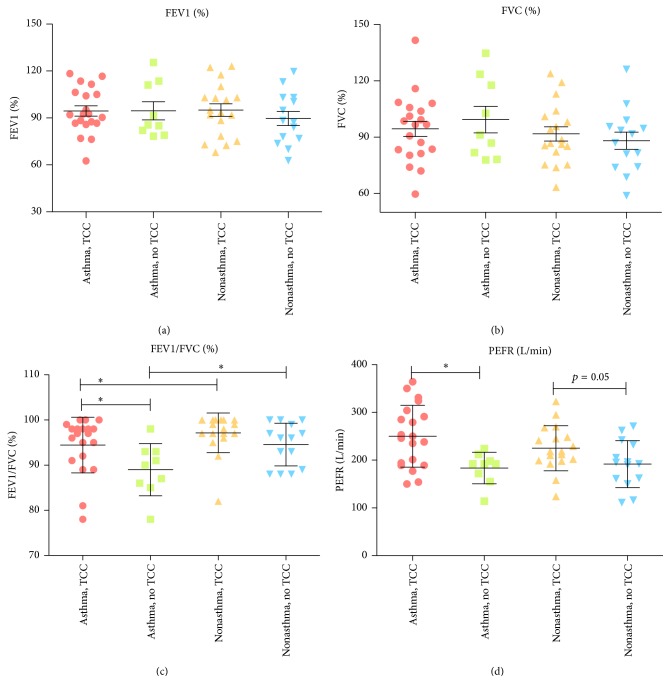
Comparison of pulmonary function between 4 groups after 3 months. *∗* indicates significant difference (*p* < 0.05).

The demographic and baseline values of outcome variables at the study entry of subjects are given in Table 1. There were no significant differences in age, gender, anthropometric measurement, and pulmonary function between groups at the study entry, except for the baseline FeNO levels (Table 1 and Figures 4 and 3(a)). We observed a higher level of baseline FeNO in the asthmatic non-TCC group, compared with the nonasthmatic non-TCC group (39.2 ± 14.9 versus 26.6 ± 17.4, *p* = 0.034) (Figure 3(a)). The levels of FEV1/FVC and PEFR increased, while the level of FeNO decreased significantly after TCC exercise for 3 months in both asthmatic and nonasthmatic TCC groups but not in either of the asthmatic or nonasthmatic non-TCC groups (Figures 2(c), 2(d), and 3(c)). There were significantly higher FEV1/FVC levels after 3 months in the asthmatic TCC group compared with the asthmatic non-TCC group (Figure 5). Levels of FEV1 significantly increased only in the asthmatic TCC group after TCC exercise (*p* = 0.03) (Figure 2(a)). There was no significant improvement in FVC after 3 months of TCC exercise for either group (Figure 2(b) and Table 3).

**Figure 6:**
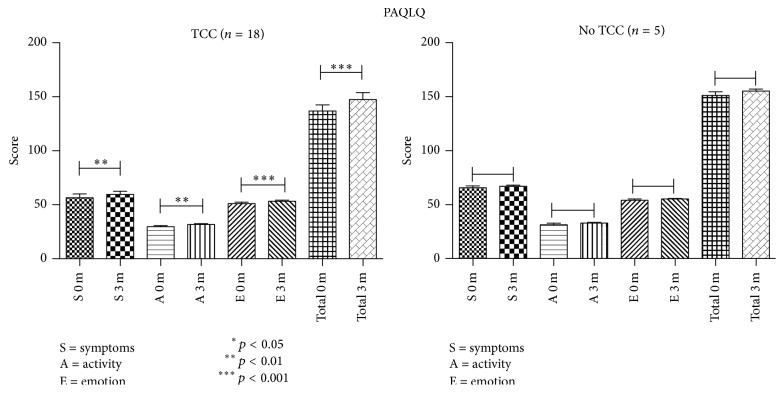
Pediatric Asthma Quality of Life Questionnaire scores in asthmatic children.

The PAQLQ scores (total scores and score in each domain) showed significant improvement over the 12-week study period only in the asthmatic TCC group (*p* = 0.0004) (Table 2 and Figure 6). Multiple linear regression models revealed that participation in TCC significantly contributed to improvements in pulmonary function (FEV1, FEV1/FVC, and PEF) and FeNO after adjusting for age, gender, body height, body weight, and baseline values of pulmonary function or FeNO (Table 4). During the study period, none of the asthmatic children had an acute asthma attack or received medication changes for asthma.

## 4. Discussion

This prospective study has demonstrated that participation in 12 weeks of TCC exercise resulted in a significant improvement of pulmonary function and FeNO (indicator of airway inflammation) in asthmatic children, mostly with mild intermittent severity. Such improvement in lung function was also observed in nonasthmatic children. However, quality of life for asthmatic children also significantly improved after TCC exercise, as assessed by the PAQLQ scores in each domain, namely, asthmatic symptoms, activity limitation, and emotional function. Our previous study showed a similar result of improvement in pulmonary function after TCC for asthmatic children [14]. The favorable effects of TCC on pulmonary function and quality of life observed in this study are consistent with the findings in a recent meta-analysis demonstrating that exercise training may improve asthma symptoms, quality of life, exercise capacity, bronchial hyperresponsiveness, exercise-induced bronchoconstriction, and lung function (FEV1) in asthmatic patients [25]. Other reviews support the findings that physical training could improve cardiorespiratory fitness and quality of life but contradict each other on pulmonary function (PEF improved but not FEV1% and FVC) in asthmatic children [26, 27].

Considering the benefits, physical exercise is generally recommended as a supplementary therapy for asthma [25–27]. However, the recommendation is conditional on controlling the intensity as a safeguard against exercise-induced bronchospasm [5, 28]. High-intensity physical activity not only carries the risk of inducing bronchospasm but also may aggravate the side effects of sympathomimetic agents and/or corticosteroids, which are taken to reduce symptoms of airway inflammation or alleviate bronchoconstriction. Side effects include increased predisposition to stress-related cardiovascular complications like arrhythmia and seizures [29]. TCC, as an exercise of moderate intensity, obviates the aforementioned risks associated with high-intensity exercise.

To assess airflow obstruction, we measured FEV1/FVC ratio to assess airflow limitation, which is widely used in the diagnosis of obstructive and restrictive lung disease [30]. FEV1/FVC is strongly associated with asthma severity, even after adjusting for percentage of predicted normative value (FEV1%) [31]. However, studies of airway inflammation by FeNO levels in asthmatic children are scarce [27]. At the time of writing, our study is the first to demonstrate the improvement of airway inflammation by FeNO levels in asthmatic children.

FeNO is derived from the action of inducible nitric oxide synthase expressed by the airway epithelium. FeNO is increased during asthma attack and correlates with airway eosinophilia [32]. There is a large variation in FeNO levels between individuals, which may reflect the natural heterogeneity in baseline epithelial nitric oxide synthase activity and/or the contribution of other noneosinophilic factors to epithelial nitric oxide synthase activity. The upper limits of normal FeNO in Asian children depend on age, from 21 ppb in young children to 39 ppb in adolescents [33]. FeNO levels in healthy children depend on several non-disease-related factors, such as age, gender, height ethnicity, genetics, self-reported atopy, allergic sensitization, total IgE, time of testing, infections, a nitrate-rich diet, exercise, smoking, ambient nitric oxide, time of day and season, and environmental pollution [18, 34, 35]. Hence, we instructed participants to follow a low-nitrate diet prior to the measurement and to perform the exam in the morning at the same time prior to exercise in order to minimize possible confounding factors [32]. The effect of TCC on FeNO in children without asthma is unexpected and suggests a general effect of TCC on the FeNO level of school children. There were several studies which disclosed that FeNO level decreases during and after exercise in nonasthmatic children [36, 37]. Our study is the first to reveal that FeNO level also decreases in nonasthmatic children after 12 weeks of TCC exercise. The mechanism of decreasing FeNO level by TCC exercise is unclear and further study is needed.

A number of limitations to our study are worth noting. First, we only enrolled children with mild intermittent asthma in our study. The effect of TCC exercise on children with moderate-to-severe asthma remains unknown. Second, the sample size was relatively small due to the reluctance of school students and parents to participate to in our study. Third, our study subjects participated in the TCC class for 1 hour once a week for 3 months and were instructed to practice TCC at home by following the provided video recordings. The compliance to our scheduled exercise at home was yet to be determined. The question of the ideal intensity or frequency of TCC exercise for asthmatic children considering different severity needs to be answered. Moreover, our study was not a randomized controlled trial and biases may exist such as allocation bias because the intervention of TCC was distributed only by the wills of participants and their parents and bias in assessing outcomes because the questionnaire is relatively subjective. Those who participated in 12-week TCC exercise may expect themselves to get better quality of life afterwards. Therefore, the results could represent regression to the mean.

Other confounding factors might also be present. We noticed higher body mass index (BMI) (21.12 ± 5.73) in asthmatic children without TCC exercise (Table 1). 44.4% of them reached overweight status according to their age reference (normal value is 15.4–20.3 in Taiwan). It is difficult to determine whether participation in TCC exercise was influenced by being overweight, by the reluctance to exercise, or by fear of exercise-induced bronchospasm. Many studies have revealed the impact of obesity on asthma patients and recommended a weight control program for obese asthmatics [38–40]. Following exercise, BMI and lipid profiles improved in overweight children, and those with asthma have also benefited from exercise [41]. The impact of TCC exercise on obese or overweight children needs further investigation.

## 5. Conclusions

In conclusion, our study provides pilot evidence that choosing TCC as an alternative form of exercise is beneficial for asthmatic children. The results indicated that 12 weeks of Tai-Chi-Chuan could improve the pulmonary function, decrease airway inflammation, and improve quality of life in children with mild asthma. It also improved pulmonary function and decreased FeNO level in nonasthmatic children. Further studies are needed to evaluate the effect of TCC on children with moderate-to-severe asthma.

## Figures and Tables

**Table 1:** Demographic and baseline values of outcome variables.

	Asthma, TCC	Asthma, no TCC	Control, TCC	Control, no TCC	*p* value
	(*n* = 20)	(*n* = 9)	(*n* = 18)	(*n* = 14)	
Age, years (mean ± SD)	10.55 ± 0.27	10.33 ± 0.53	10.11 ± 0.21	10.57 ± 0.17	0.47
Males, *n* (%)	12 (60)	4 (44)	7 (39)	6 (43)	0.58
Females, *n* (%)	8 (40)	5 (56)	11 (61)	8 (57)	

*Anthropometric measurement*					

Height, cm (mean ± SD)	130 ± 4.8	135 ± 2.58	142.3 ± 8.03	144.79 ± 7.44	0.68
Weight, kg (mean ± SD)	36 ± 9.27	38.6 ± 10.86	35.86 ± 6.22	38.36 ± 10.36	0.95
BMI (mean ± SD)	17.95 ± 3.4	21.12 ± 5.73	17.64 ± 2.49	18.1 ± 3.64	0.93

*Pulmonary function*					

FVC (% of predicted)	92.4 ± 14.9	96.81 ± 20.5	93.2 ± 14.8	90.6 ± 15.2	0.95
FEV1 (% of predicted)	89.2 ± 9.1	94.0 ± 17	92.7 ± 15.0	90.6 ± 15.1	0.86
FEV1/FVC (%)	91.3 ± 7.9	91.1 ± 6.7	93.3 ± 6.4	93.4 ± 7.6	0.65
PEFR (L/min)	194.1 ± 59.6	186.3 ± 35.0	197.7 ± 44.7	189.1 ± 44.3	0.97
*FeNO*	57.4 ± 30.7	39.2 ± 14.9	40.2 ± 25.1	26.6 ± 17.4	**0.02**

*Pediatric Asthma Quality of Life Questionnaire (PAQLQ)*					
	(*n* = 18)	(*n* = 5)			

Total score	137.2 ± 24.01	151 ± 7.61			0.28
Symptom score	56.44 ± 15.18	65.6 ± 3.65			0.18
Activity limitation score	29.78 ± 4.29	31.4 ± 3.36			0.52
Emotional function score	51.06 ± 6.12	54 ± 2.92			0.30

BMI, body mass index; FEV1, forced expiratory volume in 1 second; FVC, forced vital capacity; PEFR, peak expiratory flow rate; FeNO, fractional exhaled nitric oxide. *p value* was obtained by Kruskal-Wallis test or Mann–Whitney *U* test or depending on the type of variables. All values are expressed as mean ± SD.

**Table 2:** Pediatric asthma quality of life score.

	Subgroup	0 wk	12 wk	*p* value
Symptoms	TCC	56.44 ± 15.18	59.61 ± 11.97	**0.0051**
	No TCC	65.6 ± 3.65	67 ± 2.55	0.27

Activity limitation	TCC	29.78 ± 4.29	31.72 ± 3.44	**0.0018**
	No TCC	31.4 ± 3.36	32.8 ± 1.48	0.17

Emotional function	TCC	51.06 ± 6.12	53.17 ± 4.42	**0.001**
	No TCC	54 ± 2.92	55.2 ± 1.3	0.5

Total quality of life	TCC	137.2 ± 24.01	147.4 ± 27.13	**0.0004**
	No TCC	151 ± 7.61	155.2 ± 4.2	0.10

TCC (*n* = 18); no TCC (*n* = 5). All values are expressed as mean ± SD. *p values* were obtained by comparison with the 0 wk value via Wilcoxon matched-pairs signed-rank test in the Asthma group.

**Table 3:** Results of pulmonary function and fractional exhaled nitric oxide (FeNO).

Parameters	Group	Subgroup	0 wk	12 wk	*p* value
FEV1 (% of predicted)	Asthma	TCC	89.2 ± 9.1	94.5 ± 14.6	**0.03**
		No TCC	94.0 ± 17	94.6 ± 17.4	0.57
	Nonasthma	TCC	92.7 ± 15.0	95.1 ± 17.1	0.44
		No TCC	90.6 ± 15.1	89.6 ± 16.8	0.65

FVC (% of predicted)	Asthma	TCC	92.4 ± 14.9	94.4 ± 18	0.32
		No TCC	96.81 ± 20.5	99.4 ± 21.3	0.09
	Nonasthma	TCC	93.2 ± 14.7	93.2 ± 14.8	0.62
		No TCC	90.6 ± 15.2	88.1 ± 17.1	0.16

FEV1/FVC	Asthma	TCC	91.3 ± 7.9	94.5 ± 6.2	**0.016**
		No TCC	91.1 ± 6.7	89 ± 5.8	0.07
	Nonasthma	TCC^*∗*^	93.3 ± 6.4	97.2 ± 4.4	**0.026**
		No TCC^*∗*^	93.4 ± 7.6	94.6 ± 4.7	0.46

PEFR	Asthma	TCC	194.1 ± 59.6	250.2 ± 64.7	**<0.0001**
		No TCC^*∗*^	186.3 ± 35.0	183.4 ± 32.9	0.55
	Nonasthma	TCC	197.7 ± 44.7	225.0 ± 47.1	**0.0007**
		No TCC	189.1 ± 44.3	191.8 ± 49.2	0.52

FeNO	Asthma	TCC	57.4 ± 30.7	35.6 ± 21.6	**<0.0001**
		No TCC	39.2 ± 14.9	36.1 ± 13	0.40
	Nonasthma	TCC^*∗*^	40.2 ± 25.1	30.6 ± 21.7	**0.002**
		No TCC^*∗*^	26.6 ± 17.4	31 ± 24.4	0.10

All values are expressed as mean ± SD. All the comparisons were obtained by paired *t*-test with 0 wk values or Wilcoxon matched-pairs signed-rank test depending on the distribution of the data. *p* values were obtained from the difference among groups. ^*∗*^Data are not assumed to be Gaussian distribution.

**Table 4:** Multiple linear regression analysis.

Dependent variables	Entered variable(s)	Beta	*p* value
ΔFEV1 (% of predicted)	Weight	0.53	0.000
	Height	−0.369	0.010
	TCC	0.296	0.014

ΔFEV1/FVC	Baseline FEV1/FVC (%)	−0.651	0.000
	TCC	0.323	0.001
	Asthma	−0.239	0.013

ΔPEF	TCC	0.514	0.000
	Asthma	0.216	0.049

ΔFeNO	Baseline FeNO	−0.502	0.000
	TCC	−0.353	0.001

All the Δ values were the difference after 3 months. Variables of age, gender, asthma, TCC, body height, body weight, baseline levels of lung function parameters, or FeNO were included in the multiple linear regression analysis.
